# Use of scanning electron microscopy in the cochlea of guinea pigs

**DOI:** 10.1016/j.bjorl.2018.11.008

**Published:** 2019-01-07

**Authors:** Luiz César Nakao Iha, Oswaldo Laércio Mendonça Cruz

**Affiliations:** Universidade Federal de São Paulo (Unifesp), Escola Paulista de Medicina (EPM), São Paulo, SP, Brazil

**Keywords:** Electron microscopy, Inner ear, Cisplatin, EDTA, Microscopia eletrônica, Ouvido interno, Cisplatina, EDTA

## Abstract

**Introduction:**

The use of electron microscopy in the study of the inner ear has allowed us to observe minute details of the hair cells, especially in ototoxicity studies; however, the preparation of this material is a difficult and delicate task. In an attempt to simplify the handling of these materials, two agents, toluidine blue and ethylenediamine tetra-acetic acid were tested, in addition to the elimination of osmium tetroxide during the preparation of albino guinea pig cochleae. We also tested the applicability of these methodologies in an ototoxicity protocol.

**Objective:**

To verify the quality of the images obtained with and without the use of ethylenediamine tetra-acetic acid, toluidine blue and osmium tetroxide in the preparation of cochleae of albino guinea pigs for the scanning electron microscopy.

**Methods:**

Three groups of cochleae were used. In Group 1, 10 cochleae were prepared with the usual methodology, dissecting the optical capsule without decalcification and using osmium tetroxide as a post-fixative agent. In Group 2, we prepared 10 cochleae decalcified with ethylenediamine tetra-acetic acid, injecting toluidine blue in the endolymphatic space to facilitate the identification of the organ of Corti. In Group 3, we used 4 cochleae of guinea pigs that received 3 doses of cisplatin (7.5 mg/kg, D1–D5–D6), two prepared according to the methodology used in Group 1 and two with that used in Group 2. Scanning electron microscopy images were obtained from the organ of Corti region of the basal turn of each cochlea.

**Results:**

The organ of Corti was more easily identified with the use of toluidine blue. The dissection of the cochlea was more accurate in the decalcified cochleae. The quality of the images and the preservation of the organ of Corti obtained with the two methodologies were similar.

**Conclusion:**

The proposed modifications resulted in images of similar quality as those observed using the traditional methodology.

## Resumo

**Introdução:**

O emprego da microscopia eletrônica no estudo da orelha interna permitiu observar detalhes minuciosos das células ciliadas especialmente em estudos de ototoxicidade. Entretanto, o preparo desse material é trabalhoso e delicado. Para simplificar a manipulação desses materiais, testou-se o uso de dois agentes, azul de toluidina e ácido etilenodiamino tetra-acético, além da retirada do tetróxido de ósmio na preparação de cócleas de cobaias albinas. Testamos também a aplicabilidade dessas metodologias em um protocolo de ototoxicidade.

**Objetivo:**

Verificar a qualidade das imagens obtidas com e sem o uso de ácido etilenodiamino tetra-acético, azul de toluidina e tetróxido de ósmio na preparação de cócleas de cobaias albinas para a microscopia eletrônica de varredura.

**Método:**

Foram utilizados três grupos de cócleas. No Grupo 1 preparou-se 10 cócleas com a metodologia usual, dissecando a cápsula ótica sem descalcificação e utilizando tetróxido de ósmio como pós-fixador. No Grupo 2 preparamos 10 cócleas descalcificadas com ácido etilenodiamino tetra-acético, injetando azul de toluidina no espaço endolinfático para facilitar a identificação do órgão de Corti. No Grupo 3 utilizamos 4 cócleas de cobaias que receberam 3 doses de cisplatina (7,5 mg/kg, D1–D5–D6), duas preparadas com a metodologia do Grupo 1 e duas com a do Grupo 2. Foram obtidas imagens da microscopia eletrônica de varredura da região do órgão de Corti do giro basal de cada cóclea.

**Resultados:**

O órgão de Corti foi mais facilmente identificado com o azul de touidina. A dissecção da cóclea foi mais precisa nas cócleas descalcificadas A qualidade das imagens e a preservação do órgão de Corti obtidas com as duas metodologias foi similar.

**Conclusão:**

As modificações propostas resultaram em imagens de qualidade similar as observadas com o uso da metodologia tradicional.

## Introduction

The use of optical microscopy to study biological tissues is of utmost importance and was used by Alfonso Corti to perform the first detailed histological observations of the labyrinth organs, describing the spiral ganglion, the basilar membrane, the inner and outer hair cells and the tectorial membrane.^1^ But our knowledge about the inner ear increased even further when cochlear structures were evaluated by electron microscopy.

The electron microscope was invented in 1931 by Ernst Ruska and uses an electron beam to generate the images. As the wavelength of this beam is approximately 100,000 times smaller than that of the visible light spectrum, electron microscopy has a much higher resolution power than optical microscopy, allowing a closer observation of the cochlear microstructure.^2^

One of the modalities of electron microscopy is Scanning Electron Microscopy (SEM), in which the electron beam travels along the surface of the studied material, generating a three-dimensional image of this material. This characteristic makes SEM particularly interesting in assessing the integrity of hair cells in situations of internal ear damage, as well as in studying ototoxicity.

Ototoxicity is the result of a substance causing damage to the inner ear, with the capability of causing a functional alteration.^3^ The most often studied experimental protocols for ototoxicity are those mediated by aminoglycoside antibiotics and cisplatin^4^, ^5^, ^6^, ^7^, ^8^ in which the cochlea undergoes a type of processing that includes fixation with glutaraldehyde, removal of the otic capsule, post-fixation with osmium tetroxide, dehydration in ethanol and CO_2_ critical point and finally receiving a thin coating of metal (usually gold or platinum). However, in previous studies carried out in our service^9^, ^10^ we observed that the preservation of the organ of Corti was not always ideal, with occasional mechanical focal lesions in the region of the hair cells resulting from the removal of the otic capsule.

In an attempt to better preserve the organ of Corti and to simplify the dissection and processing of this material, we designed a model in which we modified the preparation of albino guinea pig cochleae for the SEM using Ethylenediamine Tetra-Acetic acid (EDTA) and Toluidine Blue (TB) to decalcify the otic capsule, facilitating the identification of the hair cell region and allowing a more delicate dissection of the capsule. We compared the quality of the images obtained with a group of cochleae processed in the usual manner. In this new model, we also tested the elimination of osmium tetroxide to simplify the process and avoid the need to use laminar flow when handling the material. In a second stage, we applied the two methodologies (the usual and the new technique) to the cochleae of guinea pigs submitted to an ototoxic protocol to assess image quality and applicability of this new method in this type of experimental protocols.

The aim of this study is to assess the quality of the images obtained with and without the use of EDTA, toluidine blue and osmium tetroxide when preparing cochleae of albino guinea pigs for scanning electron microscopy evaluation.

## Material and methods

Twelve healthy female albino guinea pigs (*Cavia porcellus*), with present Preyer reflexes, weighing between 350 and 450 g and approximately 3 months of age were studied. The animals were kept in cages, at stable temperature between 21 and 22 °C, with 12-h light–dark cycles and free access to food and water. The project and all its procedures were financed by the researchers and approved by the Research Ethics Committee of our institution, under number CEP1625/04.

The animals were divided into three groups.

### Group 1 – customary preparation

Five guinea pigs (10 cochleae), with no previous ototoxic medications and with present Preyer reflexes, were anesthetized with intramuscular injections of xylazine (10 mg/kg) and ketamine hydrochloride (40 mg/kg), and subsequently euthanized with an intracardiac injection of potassium. The animals were rapidly decapitated, and a medial sagittal craniotomy was performed, separating the right and left hemicranias. Subsequently, the temporal bones were isolated by removing the remaining bony portions of the cranial structure.

The mastoid bulla was opened, thereby exposing the cochlea. Under microscopic view, a fenestration was made at the cochlear apex with a micro lancet, which was used as an opening for infiltration with 2.5% glutaraldehyde fixing solution in 0.1 M cacodylate buffer, pH 7.2, through the round window and, afterwards, the material was immersed in the same fixative solution for 24 h, stored in a refrigerator at 4 °C. After this time, two 1-h washes were performed, followed by an overnight rest in the buffer solution for complete removal of the fixative agent.

Subsequently, the remainder of the otic capsule was carefully removed and the modiolus was extracted together with the organ of Corti, with this material being submitted to dehydration in increasing concentration of ethanol solutions (50%, 70% and 90%) for 30 min each, followed by three 30-min immersions in 100% ethanol and then submerged in 2% osmium tetroxide, waiting 15 min to complete the post-fixation step. Then the drying was carried out using critical point drying equipment (Balzers-CPD 030), in which the samples were submitted to several baths in liquid CO_2_, which removes all the water and ethanol still present in the samples.

This final specimen was fixed and mounted on an adequate support using conductive paste coated with a 25–50 mm thick gold layer (Balzers SCD 050 – Sputter Coater) and visualized on a JEOL scanning microscope, model 5300.

A photographic field of the basal cochlear turn was analyzed in order to verify the sharpness of the obtained image for comparison with the other groups, as well as the preservation of the organ of Corti.

### Group 2 – modified technique

Five guinea pigs (10 cochleae) were submitted to the same preparation as in Group 1; however, after the 12-h period in the buffer solution to remove the fixative agent, the material was submerged in a 10% EDTA solution, also for a 12-h period, but the post-fixation step with osmium tetroxide was omitted. Toluidine blue dye was used for better identification and preservation of the organ of Corti, utilizing a 0.1% solution, which was injected into the endolymphatic space of the cochlear basal turn through the decalcified otic capsule utilizing a syringe and needle kit (8.0 × 0.3 mm) used for insulin injections.

Using a N. 11 scalpel blade and micro-scissors for otologic surgery, the otic capsule was removed, and the modiolus was then removed together with the organ of Corti.

After the material was submitted to alcohol and critical-point dehydration, mounted in an adequate support and coated with a gold layer, a lower cochlear turn field was photographed.

### Group 3 – use of the two techniques in a cisplatin-mediated ototoxicity protocol

Two guinea pigs were submitted to three doses of 7.5 mg/kg of cisplatin following the D1–D5–D6 schedule (applications on the first day, and on the fourth and fifth subsequent days), a protocol standardized at our service that produces a proven alteration in functional hearing tests and damage to the outer hair cells. On the day following the last cisplatin application, the guinea pigs were euthanized, and one of the cochleae were submitted to the Group 1 preparation protocol (Subgroup 3a) and the other cochlea to the Group 2 protocol (Subgroup 3b). During the follow-up period, otoscopic examinations were carried out to rule out an inflammatory state of the middle and outer ear.

## Results

### Group 1

**Figure 1:**
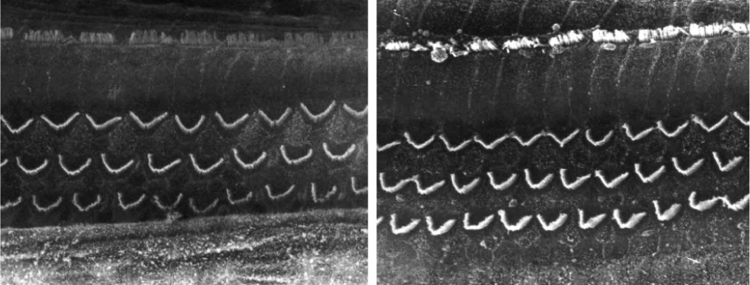
Photographs of the organ of Corti region of guinea pigs from Group 1 (use of Osmium tetroxide, without EDTA).

**Figure 2:**
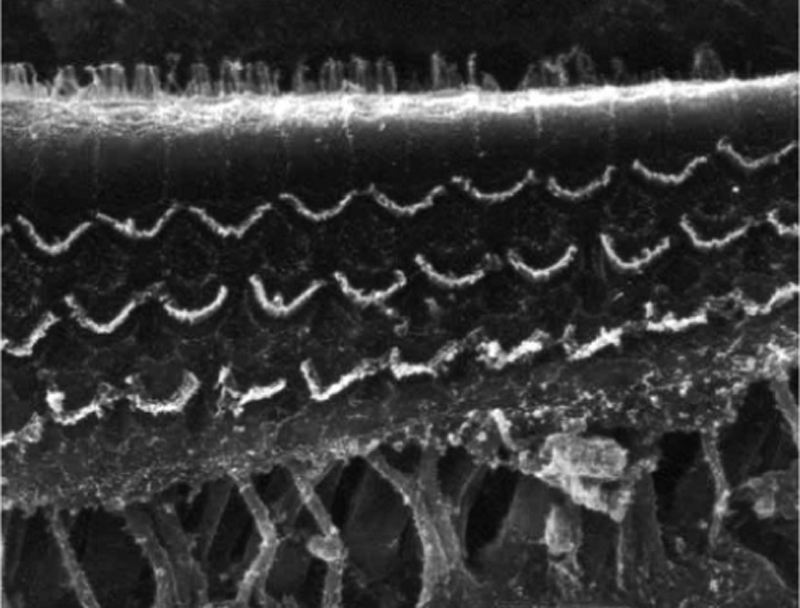
Photograph of the organ of Corti region of a guinea pig from Group 1 (use of Osmium tetroxide, without EDTA), showing the presence of a fracture next to the outer hair cells.

The images obtained in the photos of the cochleae in this group showed excellent image quality, allowing us to easily verify the presence of both the inner and outer hair cells, as shown in Fig. 1. The level of detail was adequate not only to verify the presence of hair cells, but also the integrity and position of their stereocilia. Some cochleae showed lesions in the region near the hair cells (Fig. 2). We did not find any evidence of hair cell lesion due to the procedures of the preparation of this material for the SEM.

### Group 2

**Figure 3:**
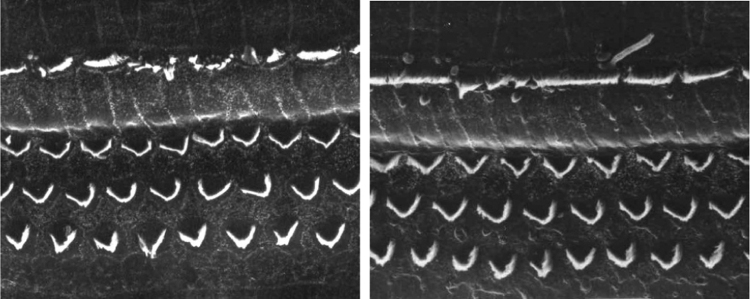
Photographs of the organ of Corti region of guinea pigs from Group 2 (use of EDTA and toluidine blue, without osmium tetroxide).

**Figure 4:**
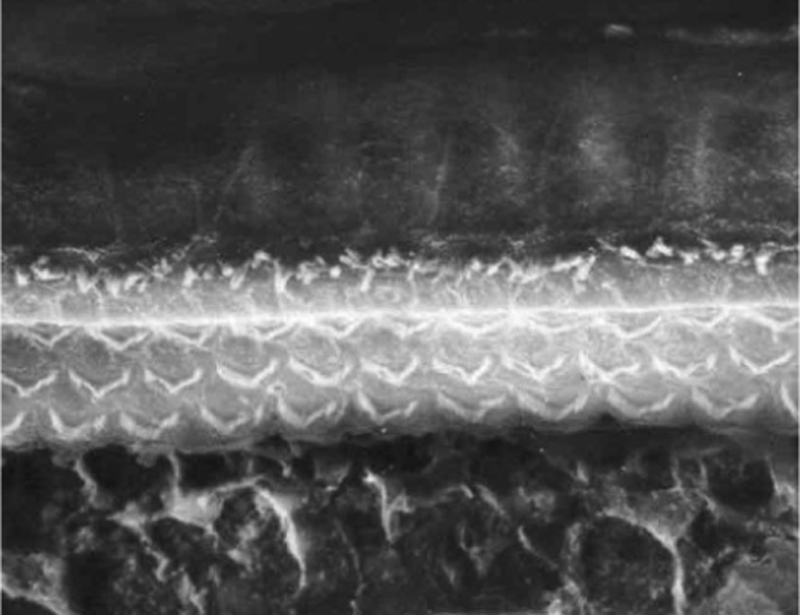
Photograph of the organ of Corti region of a guinea pig from Group 2 (use of EDTA and toluidine blue, without osmium tetroxide), with the presence of a fracture next to the outer hair cells.

There was no perceptible difference regarding the image quality of the photos obtained in this group relative to those obtained in Group 1, and it was possible to clearly observe the presence and integrity of the hair cells and their stereocilia (Fig. 3). Similar to the previous group, we observed some lesions in the region close to the hair cells (Fig. 4). In this group, we also did not observe any hair cell lesions that could indicate that the methodology interfered with hair cell preservation.

### Groups 3a and 3b

**Figure 5:**
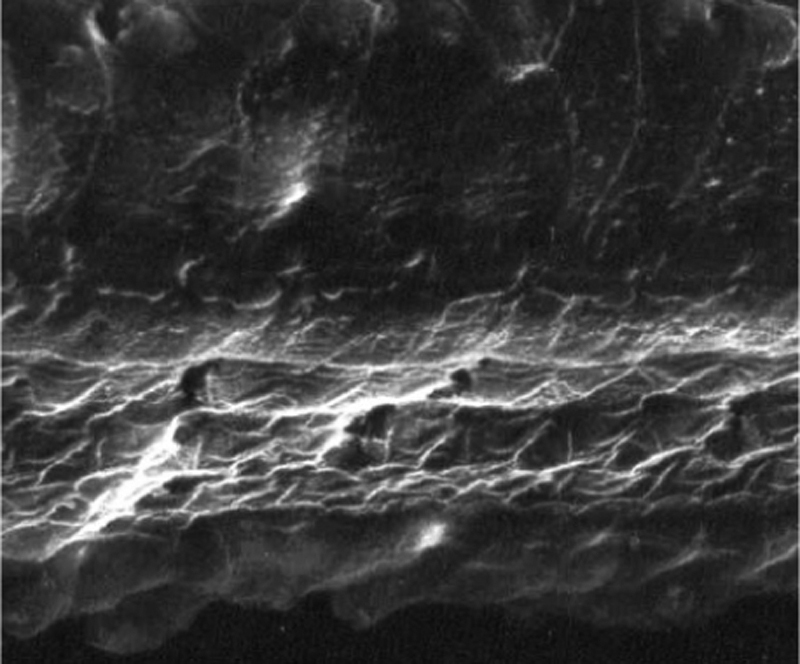
Photographs of the organ of Corti region of guinea pigs from Groups 3a and 3b.

The two groups showed images with similar resolution quality, which allowed the adequate evaluation of the organ of Corti to demonstrate hair cell lesions (Fig. 5).

## Discussion

Our anatomical knowledge was improved with the advent of optical microscopy and electron microscopy.

Optical microscopy is still the most frequently used for the study of biological materials due to its lower cost and the use of simpler equipment for its performance. Nevertheless, it is undeniable that EM attains a level of detail that makes it ideal for the study of some materials.

One of the critical points of the EM study is the preparation of the specimens for the analysis.

The preparation of the biological materials to be evaluated by EM starts with fixation (using formaldehyde, glutaraldehyde or even both^11^, ^12^), followed by post-fixation and dehydration. The post-fixation step with osmium tetroxide is routinely performed in an EM laboratory, because the electron beam used for image capture has a small penetration power and post-fixation elements containing heavy metal atoms such as uranium (uranyl acetate), lead (lead citrate) and osmium (osmium tetroxide) are required to create contrast, an extremely relevant factor for transmission electron microscopy, in which the electron beam traverses the imaging material.

However, when preparing material for the SEM, the specimens must be dehydrated, and their surface receives a metal coating, also known as sputtering, usually with gold or a gold/palladium alloy.The image captation in SEM is mainly based on the electron deflection property of this metal surface, which even makes possible to analyze the external structure of insects that have rigid exoeskeleton with no other preparation than the sputtering.^13^, ^14^ This fact led us to believe that the use of osmium tetroxide would be unnecessary to obtain images in the SEM, which was the case with the cochleae prepared with our new methodology.

The objective of trying to eliminate the use of osmium tetroxide was to avoid the high toxicity of heavy metals. Osmium tetroxide is highly toxic and extremely volatile, requiring the use of a laminar flow hood, protective goggles, apron and gloves for its handling, since exposure to this substance may cause coughing, headache, skin burns, conjunctival edema and corneal destruction, pulmonary edema, as well as its having a potential for carcinogenesis. During the handling of osmium tetroxide in the preparation of the Groups 1 and 3 cochleae in our study, we had to use laminar flow and protective gloves all the time, and yet we observed the rapid osmium tetroxide impregnation in the glove used when we opened the solution bottle.

The use of EDTA and toluidine blue aimed to allow a more delicate and precise dissection, in addition to facilitating the identification and preservation of the organ of Corti.

Toluidine blue was injected into the endolymphatic labyrinth of Group 2 cochleae and its similar specimens in Group 3b. What we observed was an immediate impregnation of the dye into the structures of the membranous endolymphatic labyrinth. After these structures were adequately identified, it was possible to be extra careful when dissecting the outer hair cell area.

EDTA is commonly used in the preparation of bone materials where extremely thin cuts are essential, but its use is not common in SEM.^15^, ^16^, ^17^ The use of EDTA in our study aimed to determine whether it would also facilitate the preparation of this material for SEM, and we found both positive and negative aspects.

On the one hand, the natural rigidity of the capsule facilitates the creation of small fracture lines that expedite its removal, but the force necessary to carry out these fractures has the potential to damage the structure to be studied.

The decalcified optical capsule of Groups 2 and 3b showed great flaccidity and malleability, requiring the use of micro scissors for their complete removal. The use of cutting tools brought greater precision to the removal of unwanted parts from the material but made the dissection a little more time-consuming.

Macroscopically, the groups in which EDTA was used, structurally seemed to be better preserved, but at the microscopic level we observed fractures similar to those found in cochleae without decalcification. This lead us to conclude that the decalcification step should be included at the discretion of the person responsible for the dissection of the cochleae, depending on the preference or need to perform a more delicate dissection.

However, the aim of this study was to verify whether the quality of the images obtained with the methodologic modifications were equivalent to the usual processing method, in addition to assessing their applicability in experimental ototoxicity models.

In the normal cochlear groups (Groups 1 and 2) the images disclosed identical image definition. By using a protocol standardized in our institution, comprising three doses of 7.5 mg/kg of cisplatin, we were able to create outer hair cell lesions that were easily identified by the SEM, making it possible to find total disarray or absence of the stereocilia in the lower cochlear turns. Considering that these alterations were observed without any difficulties in all our cochlear groups, we conclude that the modifications applied to the preparation methodology used in these samples for SEM are applicable in this model of ototoxicity caused by cisplatin.

## Conclusions

The quality of the images obtained with the proposed modifications was similar to that observed when using the traditional technique.

## Conflicts of interest

The authors declare no conflicts of interest.
